# Electronic, Excitonic, and Optical Properties of Zinc Blende Boron Arsenide Tuned by Hydrostatic Pressure

**DOI:** 10.1021/acsomega.4c07598

**Published:** 2024-11-16

**Authors:** Elisangela da Silva Barboza, Alexandre C. Dias, Luis Craco, Sabrina S. Carara, Diego R. da Costa, Teldo A. S. Pereira

**Affiliations:** † Instituto de Física, Universidade Federal de Mato Grosso, 78060-900 Cuiabá, MT, Brazil; ‡ Institute of Physics and International Center of Physics, University of Brasília, Brasília 70919-970, DF, Brazil; ¶ Departamento de Física, Universidade Federal do Ceará, 60455-900 Fortaleza, CE, Brazil; § Department of Physics, University of Antwerp, Groenenborgerlaan 171, B-2020 Antwerp, Belgium; ∥ National Institute of Science and Technology on Materials Informatics, Campinas 13083, Brazil

## Abstract

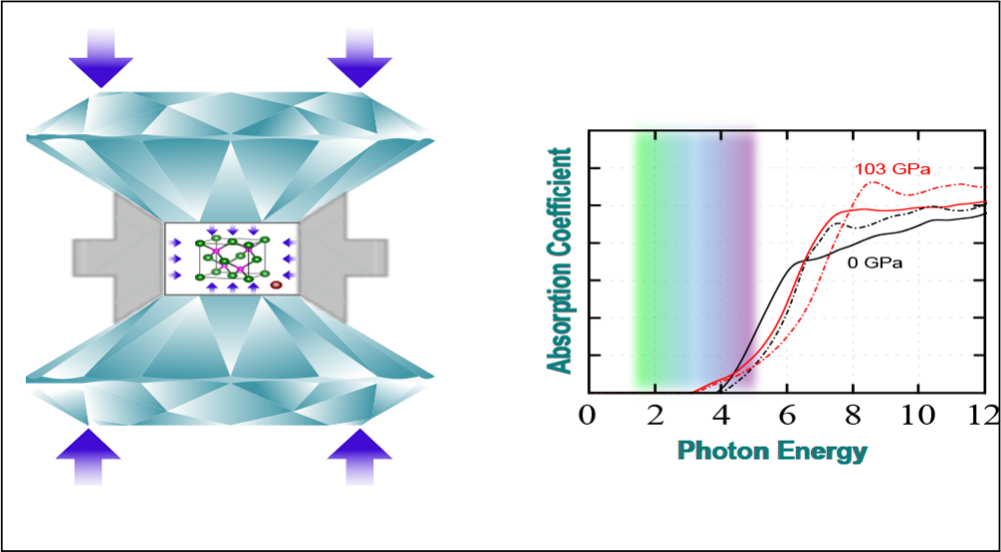

Based on first-principles calculations combined with a maximally localized Wannier function tight-binding method and the Bethe–Salpeter equation formalism, we theoretically investigate the effects of hydrostatic pressure on the electronic, excitonic, and optical properties of zinc blende boron arsenide. Our findings show: (i) a pressure-induced semiconductor-to-metallic phase transition without causing any change in the structural crystallographic ordering, (ii) a decrease in excitonic binding energy with increasing pressure as a consequence of band gap engineering, and (iii) a small excitonic response in the indirect absorption regime due to the indirect band gap.

## 1 Introduction

Cubic boron arsenide (BAs) is a semiconductor with interesting physical properties that have recently gained considerable attention.^1−5^ Bulk BAs has a zinc blende crystal structure (space group *Fm*3̅*m*) with a lattice parameter of 4.77 Å.^6,7^ Although it was synthesized back in 1958 by means of the direct reaction of boron and arsenic elements at high temperatures,^8^ only recently high-quality BAs single crystals have been grown with a low order of lattice defects.^2−4,6,9,10^ Before that, the synthesis challenges, for instance, were (i) the melting point of B being higher than the sublimation point of As, (ii) the toxicity, and (iii) the difficulties in dissolving them into common solvents, among other issues.^11^ Only in the 1970s, these difficulties have been overcome through the chemical vapor transport (CVT) method.^12^ Broadly speaking, this method has opened the possibility of obtaining pure BAs bulk crystals with higher quality, providing essential information for theoretical and experimental investigations in the search for possible future applications. Motivated thereby, Kang and co-workers reported the growth of a high-quality BAs crystal synthesized by CVT reaction without detectable defects and with an ultrahigh thermal conductivity of 1.300 W/mK at room temperature.^2,3^ Theoretical studies also highlighted BAs high thermal conductivity, which is found to be similar to that of diamond, with thermal phonon transport indicating the same value of 1.300 W/mK.^13^ From a technological perspective, heat transport from active devices requires materials with high thermal conductivity, and this makes BAs a true potential candidate for thermal energy dissipation, which is presently a major current problem in the thermal management of electronic components.^14−18^

BAs is a member of the III–V semiconductor group, such as gallium arsenide (GaAs), indium arsenide (InAs), and aluminum arsenide (AlAs),^19^ and displays an electronic band structure that resembles electronically bulk silicon (Si). It is considered to be a potential candidate for novel optoelectronic,^20−24^ photovoltaics cells, and photoelectrode applications.^17^ Earlier reports on the band gap of BAs, such as those from BAs films deposited on the basal plane of hexagonal silicon carbide substrates and sodium fluoride and silicon at 800–850 °C, suggested a direct band gap around 1.45 eV.^12^ However, these results may have been influenced by defect absorption,^12^ which could explain its lower value. Similarly, early studies on cubic and rhombohedral BAs using vapor-phase techniques reported band gaps of 1.46–1.51 eV, though these were determined from powder samples, they may also have been affected by sample quality and defects.^25^ More recent and refined experimental methodologies, such as those using spectroscopic ellipsometry combined with transmission and reflection spectroscopy, have shown that BAs has an indirect band gap of approximately 2.02 eV and a direct gap near 4.12 eV.^26^ These values are considered more reliable and align closely with our theoretical results using hybrid functionals, as discussed further. Additionally, theoretical calculations and the use of the Tauc plot for absorption spectrum optimization have yielded band gap estimates for cubic BAs around 1.835 eV,^27^ further demonstrating that variations in both experimental and theoretical results depend on the specific methodologies and approximations employed. Thus, while earlier studies reported a wide range of values, more recent experiments and the detailed theoretical investigation employed in the current study provide clearer results and a more accurate picture of the band gap nature of BAs. The indirect band gap semiconductor presents its conduction (valence) band minimum (maximum) setting along the Γ- to -*X* direction and has a *p*-band character. However, the exciton has a clear effect on the direct absorption with a binding energy of around 40 meV,^20,26,28^ leading to strong absorption of photons with energies higher than that of the indirect band gap.^26^

Recently, ref (29) reported the electronic structure reconstruction of a BAs bulk crystal under biaxial tensile strain. The authors applied first-principles calculations combined with the coherent potential approximation for disorder, unveiling strain- and disorder-induced electronic reconstruction and orbital differentiation of the BAs bulk crystal. Motivated by this, here we show how the electronic, excitonic, and optical properties of BAs can be tuned by applying hydrostatic pressure with spatial atomic deformation being applied equally in all directions. In the strain and stress context, previous experimental studies reported that the BAs bulk crystal supports very high pressures without undergoing a phase transition, also showing the role of applied pressure on thermal conductivity.^1^ Furthermore, phase transitions from the zinc blende phase to an amorphous crystal structure take place in BAs at 125 GPa and high temperatures. Interestingly, the amorphous phase persists up to 165 GPa, remaining stable when reducing pressure to normal conditions.^30,31^ Recent experimental measurements^2,4^ have confirmed the stability of bulk BAs and theoretical studies performed by Mortazavi et al.^32^ in 2021 and by Arrigoni and Madsen^33^ in 2019 on the phonon spectrum further validated such structural stability by means of first-principles calculations, showing the absence of negative frequencies in the phonon dispersion. A theoretical study^34^ on monolayer hexagonal boron arsenide reported tunable thermal conductivity by strain engineering, predicting that the thermal conductivity of a stress-free and pristine monolayer is in the order of 180.2 W/mK and that it can be substantially enhanced to 375.0 and 406.2 W/mK with only 3% of strain along the armchair and zigzag directions, respectively. In this context, we aim here to describe how hydrostatic pressure tunes the electronic, excitonic, and optical properties of the BAs zinc blende structure, based on first principle methods combined with a maximally localized Wannier function tight-binding (MLWF-TB) model and the Bethe–Salpeter equation (BSE) formalism. With this in mind, in Section 2, we discuss the theoretical details implemented by computational packages. Results and discussions are presented in Section 3 for the electronic, excitonic, and optical properties of the pressurized BAs bulk crystal. Our final remarks are pointed out in Section 4.

## 2 Computational Methods

To provide a realistic description of the electronic, excitonic, and optical properties of pressurized BAs, we performed the first-principles calculations based on Kohn–Sham density functional theory (DFT),^35^ using the Quantum Espresso (QE) package,^36^ within the projector augmented wave (PAW) method.^37^ To reveal the changes in the crystal structure and electronic properties induced by hydrostatic pressure, we applied the exchange-correlation functional in the scope of the generalized gradient approximation (GGA)^38^ proposed by Perdew–Burke–Ernzerhof (PBE).^39^ In our study, we performed crystallographic structural optimizations at each different pressure. To ensure that the symmetry of the BAs crystal structure is preserved under hydrostatic pressure, we employed the “vc-relax” option within the Quantum Espresso package. This method allows for the relaxation of both atomic positions and unit cell parameters while applying external pressure. We used the “cell-dynamics” flag to activate the quasi-Newton algorithm for relaxation, along with the “press” flag to apply pressure isotropically in kbar units. The “cell-dofree” variable was set to “all”, ensuring that all lattice vectors were free to relax uniformly. After relaxation, we verified that cubic symmetry was maintained by performing symmetry checks on the relaxed structure and confirmed that the pressure was applied uniformly across all directions, with no deviation from the expected isotropic contraction (see results in Section S1 of the Supporting Information^40^). These checks confirmed that the symmetry of the system remained intact throughout the computational process. Table S1 of the Supporting Information^40^ shows the optimized atomic positions and interatomic distances for each investigated BAs crystal structure subjected to pressure. In the PBE simulations, we assumed a cutoff energy of 50 Ry, with a convergence criterion of 10^–8^ Ry and a 16 × 16 × 16 **k**-mesh, generated with the Monkhorst–Pack method.^41^ Moreover, due to self-interaction errors presented in PBE, which in turn underestimate the band gap size,^42,43^ we employed the separated ranged hybrid exchange-correlation functional proposed by Heyd–Scuseria–Ernzerhof (HSE06).^44−46^ In our framework, the Fock-exchange is calculated considering an 8 × 8 × 8 **k**-mesh, and the remainder of the Kohn–Sham Hamiltonian is evaluated within the same PBE **k**-mesh. In the HSE06 simulations, a cutoff energy of 50 Ry was used. A denser *k*-point mesh or extrapolation allows the achievement of more precise excitonic calculations, whose choice was adopted according to the computational resources available for this study and the convergence of the results. The HSE06 band structure is obtained using an MLWF-TB Hamiltonian, directly extracted from the DFT HSE06 simulation, using the Wannier90 package^47^ and considering *s-* and *p-*orbital projections from B and As chemical species.

The excitonic and optical properties were investigated by means of the WanTiBEXOS code^48^ within the scope of the independent particle approximation (IPA), which does not take into account excitonic quasi-particle effects. In contrast to other methods, the BSE explicitly includes electron–hole interaction effects.^49,50^ These physical properties were obtained using the MLWF-TB Hamiltonian (HSE06) to deal with the electron and hole single-particle states. The BSE was solved using the 3D Coulomb potential (V3D),^48^ with a **k**-point density of 40 Å^–1^ in each lattice vector direction, considering the lowest three conduction bands and the highest three valence bands. We also used a smearing of 0.05 eV for the optical properties to compute the absorption coefficient and refractive index at BSE and IPA levels.

## 3 Results and Discussion

**Figure 1:**
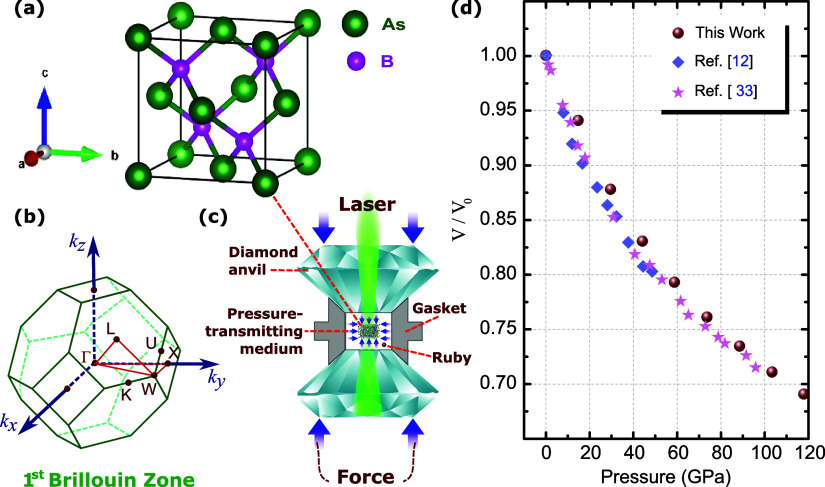
(a) BAs zinc blende crystal unit cell representation. (b) High-symmetry points and paths of the first Brillouin zone for the FCC lattice of the normal and compressed band structures. (c) Diamond anvil cell experimental setup for applying high-pressure in the bulk BAs crystal. (d) Bulk volume (*V*) dependence on pressure for the BAs crystal normalized to the value obtained under ambient pressure conditions (*V*_0_). Our results (brown spheres) are compared with experimental values taken from refs (11) and (30), showing a very good theory-experiment agreement.

BAs implemented by computational packages in this work consists of a zinc blende unit cell, as shown in Figure 1a, belonging to the space group *Fm*3̅*m* and formed by *As* (green large spheres) and *B* (pink small spheres) chemical species, with an unstrained lattice parameter of 4.77 Å.^6,7^Figure 1b shows the high-symmetry points and the paths in the first Brillouin zone used to calculate the energy bands with and without hydrostatic compression. An interesting strategy to analyze changes in the physical properties of a given material is to cause deformation through ultrahigh static pressure. For the sake of generality, we recall here that finely ground BAs powder is required in high-pressure experiments.^1,30^ The pressure vessels consist of diamond anvil cells (DACs) of the controlled displacement type. A diagrammatic sketch containing an experimental setup of a DAC for high-pressure studies is shown in Figure 1c. This experimental setup consists of a sample chamber and a metal gasket technique for hydrostatic pressure generation, i.e., transmits the same pressure over all crystal surfaces.^51^ The choice of diamond is not only due to its ultrahigh hardness but also its difficult ultrahigh phase transition and its transparency to X-rays and visible light.^52,53^ The use of small ruby crystals inside the gasket is one of the methods to determine the pressure inside the camera through ruby fluorescence. Theoretically speaking, such equally directional applied compression is the hydrostatic pressure implemented here to deform the BAs crystallographic lattice structure, as indicated by the all-directional blue arrows in Figure 1c. Furthermore, the pressure of this equipment varies from 0.1 to 400 GPa in conventional DACs.^52^ Here, the results are analyzed in the pressure range of 0–118 GPa.

First, in order to validate our theoretical approach and compare the obtained results with those already reported in the literature, we explore how the bulk volume (*V*/*V*_0_) of BAs changes under hydrostatic pressure. Such a comparison is presented in Figure 1d, where our DFT results (brown spherical symbols) demonstrate good agreement with the experimental values reported in the literature by Tian et al.,^11^ (light lilac rhombus) and by Greene et al.^30^ (magenta stars). One notices that the application of hydrostatic pressures ranging from 0 to 118 GPa induces a reduction of around 30% with respect to the original undeformed volume (*V*_0_). In addition, we also applied the PBE approach combined with the Murnaghan formalism to compute the bulk modulus, obtaining a value of 125 GPa. Such a value is close to the experimental one reported by Tian et al.^11^ of 142 GPa. Interpreting these results, along with the Vickers hardness of 22 GPa, demonstrates that BAs is a hard semiconductor. While BAs is much more brittle than materials like SiC, BN, and AlN, it is significantly more ductile than semiconductors such as Si, Ge, GaN, and GaAs. As shown by Tian and co-workers in ref (11), this behavior highlights BAs’s intermediate position between brittle and ductile materials.

### 3.1 Electronic Properties

**Figure 2:**
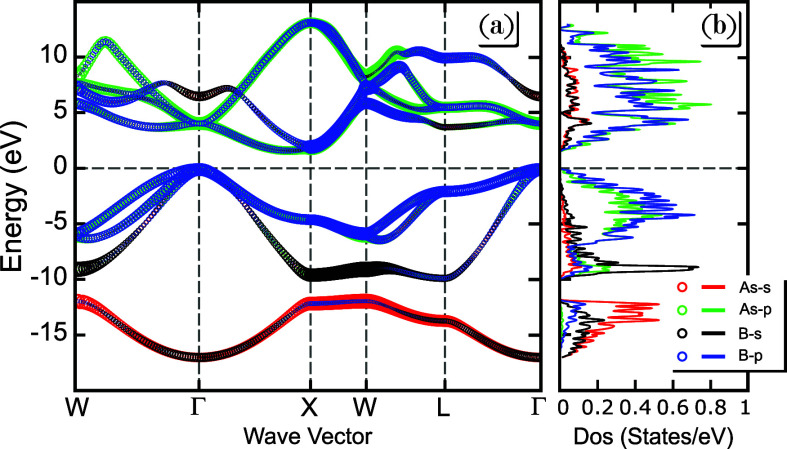
(a) Orbital-projected electronic band structure of bulk BAs at ambient pressure, presenting the different orbital-resolved contributions: As *s*-orbital (red circle), As *p*-orbital (light green circle), B *s*-orbital (black circle), and B *p*-orbital (blue circle). (b) BAs orbital-projected density of states (DOS) related to the band structure of the panel (a): As *s*-orbital (red line), As *p*-orbital (light green line), B *s*-orbital (black line), and B *p*-orbital (blue line). The Fermi energy level is set at 0 eV, and both calculations are done at the PBE level.

Before investigating the effects of deformation on the electronic properties of BAs, it is worth discussing the strain-free case, as presented in Figure 2. Additional results similar to Figure 2a are presented in Figure S8 of the Supporting Information.^40^ According to HSE06 (PBE) calculations, zinc blende BAs display an indirect band gap of 1.73 eV (1.36 eV) located in reciprocal space between Γ-*X* high-symmetry points, as shown in Figure 2a. The obtained band gap value is in agreement with the range of experimental values of 1.46–2.1 eV.^12,17,25−27^ Based on the orbital-projected energetic band structure shown in Figure 2a, it is visible that the orbital contribution majority of the valence band maximum (VBM) and the conduction band minimum (CBM) comes from the As and B *p*-orbitals. This can be confirmed by analyzing in Figure 2b and Figure S8 of the Supporting Information,^40^ the atom projected density of states (DOS) within the valence configurations 2s^2^ 2p^1^ and 4s^2^ 4p^3^ to B and As, respectively, calculated using the PBE approach in a cubic BAs bulk crystal. It should be noted that the predominant contributions for the hole and electron bands are from the B-*p* and As-*p* orbitals, whereas the other B-*s* and As-*s* orbitals contribute less significantly. Due to the nature of the strong covalent bonds, such a result depicts in summary that the valence and conduction bands are roughly equally composed of both B-*p* and As-*p* orbitals at low energies close to the Fermi Energy (*E*_F_), set here at 0–2.1 eV.

The on-site energy values for the B and As *p* orbitals are notably close, indicating a strong overlap and hybridization between these orbitals (see Table S2 of the Supporting Information^40^). This proximity suggests that neither B nor As dominates the valence band states, leading to a shared contribution from both atoms. Typically, in III–V compounds, group III elements act predominantly as cations and group V elements as anions, with the valence bands being largely derived from the anion’s *p* states. However, in BAs, the almost equal contribution from B and As *p* orbitals results in a more covalent character, which inverts the traditional cation–anion roles to some extent. This phenomenon is reflected in the valence bands being nearly equally B *p*-like and As *p*-like, a consequence of the low ionicity due to the second-row position of boron in the periodic table. As a result, the band structure shows a balanced participation of both atoms in the formation of the electronic states, leading to a more symmetric distribution of charge. This orbital configuration influences the material’s optical and electronic properties, such as its indirect band gap, and suggests that BAs occupies an unusual position among III–V semiconductors in terms of its bonding characteristics. From Table S2, one notices that the on-site energies of the arsenic (As) *p* orbitals are higher than those of boron (B) *p* ones and increases as the applied pressure amplitude increases, resulting in *B* atoms playing the role of anions, and the material becomes more ionic under pressure.

**Figure 3:**
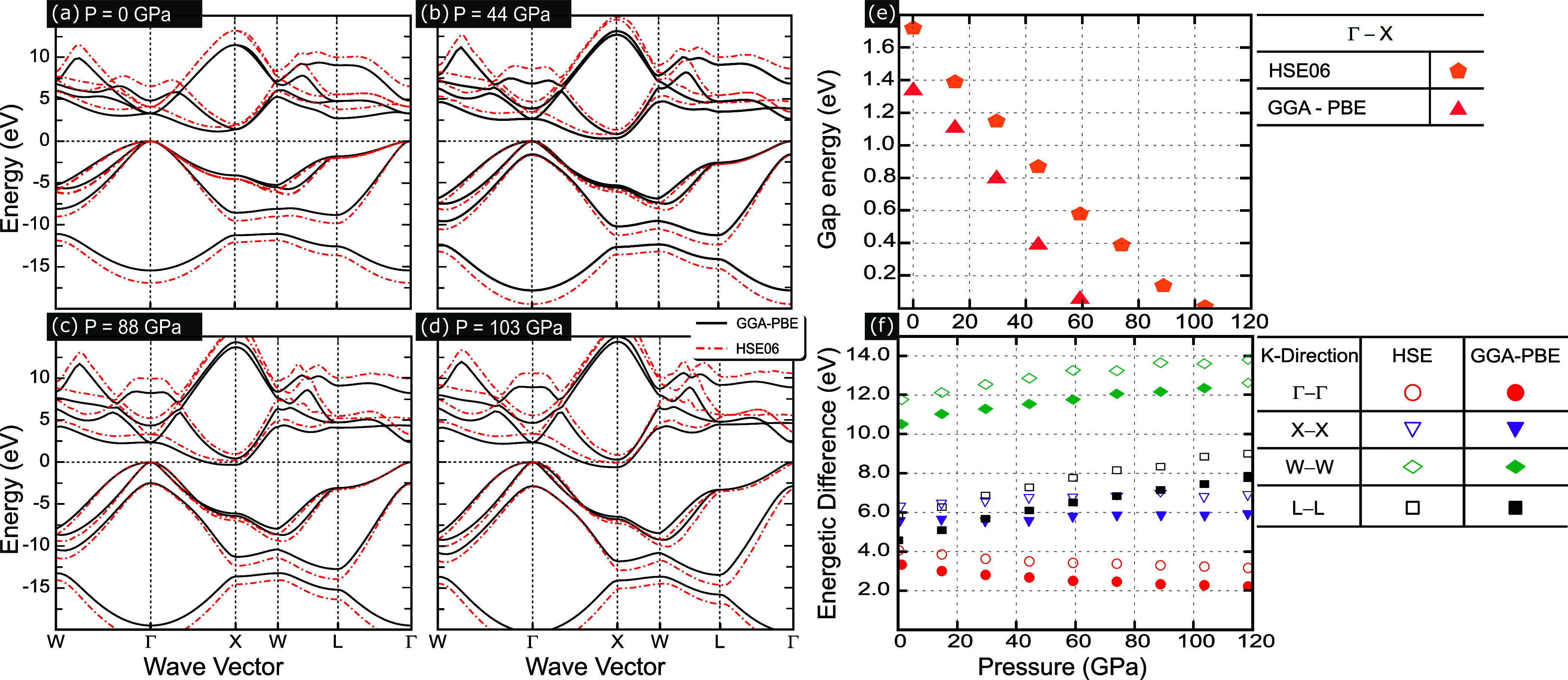
Bulk BAs electronic band structures computed within the PBE (black solid curves) and HSE06 (red dashed curves) levels of approximation at different hydrostatic pressures: (a) 0, (b) 44, (c) 88, and (d) 103 GPa. (e) Band gap energy as a function of the applied hydrostatic pressure by taking the real indirect band gap energy of the system, i.e., for an energetic difference between the VBM at Γ-point and the CBM at *X*-point, and (f) energetic difference from Γ to Γ, from *X* to *X*, and from *W* to *W* high-symmetry points.

The effect of hydrostatic pressure on the electronic band structure is shown in Figure 3a–d, with pressure magnitudes ranging from 0 to 103 GPa, obtained here within the PBE (black curves) and HSE06 (red curves) formalisms. Additional results similar to Figure 3a–d are presented in Figure S4 of the Supporting Information.^40^ First, by carefully analyzing the BAs band structure, by making an enlargement of the energy bands close to the valence bands in the vicinity of the Γ-point, one observes that valence band energies are not degenerate, as can be verified in Figure S3, which shows a zoomed-in view of the valence bands for four applied hydrostatic pressure amplitudes: (a) 0, (b) 44, (c) 88, and (d) 103 GPa. By applying hydrostatic pressure, one notices that the two valence lower-energy states stay close but do not degenerate, whereas the third state lowers its energy. It is worth mentioning that the larger the applied pressure amplitude, the smaller the volume and the closer the atoms are, leading to a larger overlap of the atomic orbitals. This is the reason why the energy bands are moved apart as the pressure value increases, i.e., the physical effect of the proximity of the atomic orbitals due to pressure resembles quantum wells’ physics when they are brought together, lifting the energetic space between the energy levels. This can be seen by comparing the energy distance of the third level for different pressure values in Figures S3 and 3a–d. All the analyzed configurations here, aiming to be more realistic, were obtained by applying hydrostatic pressure, choosing the relative atomic positions to vary when the material is under pressure, i.e., allowing the variation of the position of arsenic (As) relative to boron (B). According to Figure S2, one notices that the position of the As atoms relative to the B atoms is no longer (1/4, 1/4, 1/4) under pressure, as it should be in a zinc-blended configuration. This means that while the lattice vectors maintain the fcc cubic lattice, the atomic positions broke the cubic symmetry of the group Td, and the symmetry became rhombohedral under pressure. Thus, by allowing the atomic positions to change, it leads to symmetry breaking, which is the reason why the valence bands do not remain almost degenerate at the Γ-point. Therefore, in order to verify the emergence of symmetry breaking, leading to the break of degeneracy of the valence bands, we performed calculations by fixing the As positions at (1/4, 1/4, and 1/4) and compared them with our previously obtained results. Figures S7 and S10 of the Supporting Information^40^ confirm that by fixing the atomic positions (see red curves in Figures S7 and S10), the valence band maximum at Γ remains three-fold degenerate. Thus, our results suggest valence band degeneracy breaking is a natural response of the structure to hydrostatic pressure by alteration of the position of As. At the lowest conduction bands, one observes a degeneracy breaking at the *X*-point and along the *X*–*W* path. Moreover, the CBM at the *X*-point lowers its energy, touching the Fermi level at a pressure amplitude of 88 GPa within the PBE approximation and crossing the Fermi level at high-pressure values, as shown in Figure 3d for the 103 GPa pressure case, whereas the CBM within the HSE06 approximation touches the Fermi level for higher pressure amplitudes when compared with the PBE approach at 103 GPa.

Figure 3a–d shows a phase transition from semiconductor-to-metal behavior tunable by hydrostatic pressure, which decreases the gap energy asymptotically toward zero. It should be noted from Figures 3c,d for high pressures, a *E* = 0-band crossing occurs at the PBE level approach, leading to a half-filled conduction band. Such band gap decreasing is emphasized in Figure 3e, Figure S6 of the Supporting Information,^40^ as well as in the second column of Table 1 by the obtained band gap values using PBE and HSE06 approximations. The application of pressure causes crystal lattice contraction, which shortens the B–As bond length and, in turn, leads to a band gap that decreases as pressure increases. The observed behavior is consistent with that reported in ref (15). Interestingly, by extrapolating the data shown in Figure 3e within the PBE level of approximation, it would lead to the system presenting a semiconductor-to-metal transition at 74 GPa, and the same transition occurs starting from 103 GPa for the HSE06 functional, as shown in Table 1. Furthermore, analyzing the energetic differences associated with possible direct electronic transitions at the high-symmetry points Γ, *X*, and *W* as a function of the applied hydrostatic pressure, as shown in Figure 3f and Figure S5 of the Supporting Information,^40^ and the corresponding results in the third column (Δ*E*_Γ_) of Table 1 for the transitions Γ–Γ show that at different points of the Brillouin zone, the energetic difference between the conduction to valence bands also decreases as the full band gap energy decreases, implying a compression of such bands up to the Fermi level. However, at other points *X*–*X*, *L*–*L*, and *W*–*W*, there is a widening of the bands.

**Table 1:** Effects of External Pressure *P* on the Electronic Band Gap and the Excitonic Responses^a^

*P* (GPa)	*E*_g_ (PBE/HSE06) (eV)	Δ*E*_Γ_ (eV)	*Ex*^Γ–Γ^ (eV)	*Ex*_b_ (meV)	*Ex*_b,1_^H^ (meV)
0	1.36/1.73	4.130	4.000	133.0	23.66
15	1.12/1.40	3.899	3.830	70.0	18.35
30	0.81/1.16	3.661	3.608	53.0	18.75
44	0.40/0.88	3.496	3.448	48.0	17.92
59	0.07/0.59	3.388	3.335	54.0	19.00
74	metal/0.40	3.327	3.272	55.0	18.49
88	metal/0.15	3.306	3.244	62.0	17.93
103	metal/0.02	3.316	3.243	73.0	17.32
118	metal/metal	3.354	3.261	93.0	20.19

a Here, *E*_g_ denotes the electronic band gap obtained using PBE and HSE06 frameworks, Δ*E*_Γ_ denotes the HSE06-based Γ–Γ points energetic difference, *Ex*^Γ–Γ^ denotes the exciton state energy at the Γ point (MLWF-TB+BSE), *Ex*_b_ denotes the corresponding exciton binding energy (MLWF-TB+BSE) obtained by the difference of Δ*E*_Γ_ – *Ex*^Γ–Γ^, and *Ex*_b,1_^H^ is the ground state exciton binding energy calculated from the hydrogenic model.

### 3.2 Excitonic and Optical Properties

Once the effects on the energy band structures of the bulk BAs crystal caused by hydrostatic pressure were discussed in the previous section, we shall now investigate the consequences of such band changes on the optical and excitonic properties of bulk BAs. Let us start with excitonic effects. The fourth and fifth columns of Table 1 present the exciton state *Ex*^Γ–Γ^ of the BSE solution at the Γ-point associated with the Γ–Γ state and the corresponding exciton binding energy *Ex*_b_, respectively, for different applied hydrostatic pressures. The following column (*Ex*_b_) is calculated by the difference between the obtained results in the third column for the HSE06 electronic difference Δ*E*_Γ_ at the Γ-point and the exciton state *Ex*^Γ–Γ^, i.e., *Ex*_b_ ≡ Δ*E*_Γ_ – *Ex*^Γ–Γ^. One notices that the excitonic effects are diminished as the hydrostatic pressure magnitude increases. Table 1 shows that at 0 GPa, the exciton binding energy (*Ex*_b_) is 133 meV and decreases to 48 meV up to a pressure of 44 GPa. For higher pressures, the Γ–Γ exciton binding energy *Ex*_b_ starts to increase again, reaching 93 meV at 118 GPa; the physical reasons for this nonmonotonic behavior are still unclear and should be the objective of future investigations. Since bulk BAs is known to have an indirect band gap, one would expect a minimal excitonic effect in the indirect absorption regime, similar to the case of silicon, where the excitonic effect is also insignificant in the indirect gap regime.^54^ This is due to the small overlap of the electron and hole wave functions near the conduction and valence band edges. In light of this, and based on the results obtained for direct excitons at the Γ-point (shown in Table 1), we observe that the optical band gap renormalization shows little differences from the quasi-particle gap. This minimal difference indicates that the exciton contribution to the optical properties remains small, even under varying pressure conditions.

For comparison purposes, the latter column *Ex*_b,1_^H^ in Table 1 depicts the ground state exciton binding energy calculated from the hydrogenic-like model at the Γ-point, which is given by
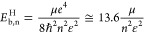
1where μ = *m*_e_*m*_h_/(*m*_e_ + *m*_h_) is the reduced effective mass for the electron–hole pair, ε is the BAs static dielectric constant, which includes both electronic and lattice contributions, and *n* is the principal quantum number of the excitonic state, *n* = 1 for the ground state. 13.6 eV is the Rydberg energy, the binding energy of a hydrogen atom. The exciton binding energies reported in Table 1, obtained using the BSE, overestimate the binding energies, which typically include only electronic screening. It computes the excitonic properties by solving the two-particle (electron–hole) equation, factoring in electron–electron and electron–hole interactions, but does not account for lattice or phonon contributions to the dielectric screening (which would lower the binding energy). This leads to an overestimation of the exciton binding energies because the true static dielectric constant in materials includes contributions from both electronic and lattice responses. In the hydrogenic model, a simpler approach often used for exciton binding energies, both electronic and lattice (ionic/phononic) contributions are included through the static dielectric constant. This model assumes that excitons behave similarly to a hydrogen atom (electron bound to a proton), where screening is more comprehensively included. The static dielectric constant used here is generally lower than what is obtained using purely electronic screening (as in BSE), which in turn reduces the binding energy (compare the fifth and sixth columns of Table 1). In essence, concerning the methods to calculate the exciton binding energies, it is relevant to summarize that the BSE formalism is more sophisticated and handles many-body effects better, but it tends to overestimate binding energies due to the omission of lattice screening, whereas the hydrogenic model compensates for this with the inclusion of lattice effects but lacks the detailed electron–hole interaction found in the BSE, as verified in Table 1.

**Figure 4:**
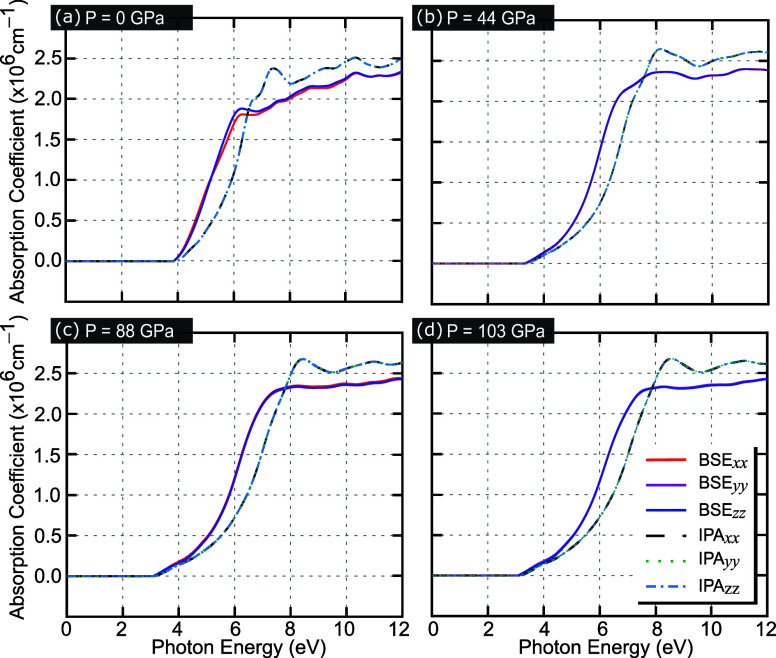
BAs absorption coefficients computed within the BSE (solid curves) and IPA (dashed curves) simulation schemes, considering the light polarization at *x*, *y*, and *z* directions for different hydrostatic pressures: (a) 0, (b) 44, (c) 88, and (d) 103 GPa.

**Figure 5:**
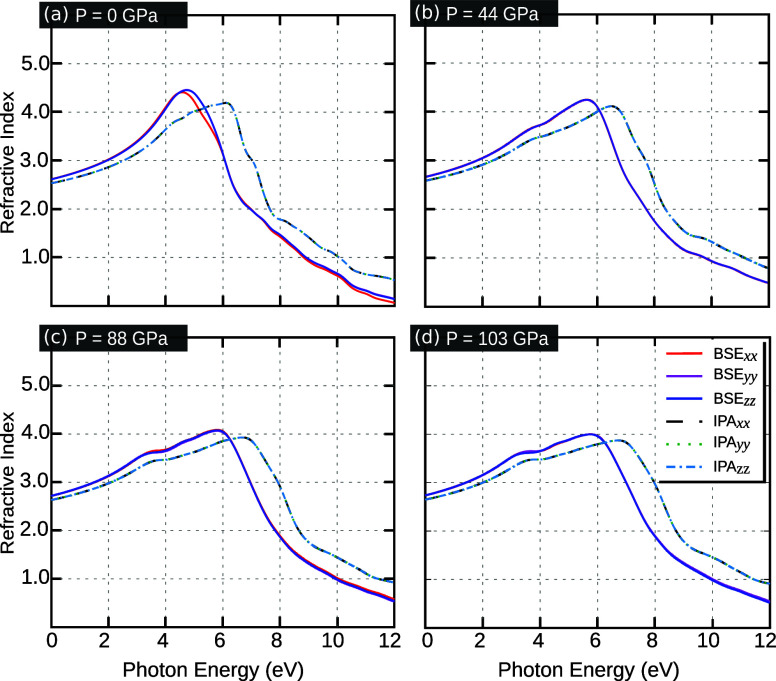
BAs refractive indexes obtained using the BSE (solid curves) and IPA (dashed and dotted curves) simulation schemes, considering the light polarization at *x*, *y*, and *z* directions for different hydrostatic pressures: (a) 0, (b) 44, (c) 88, and (d) 103 GPa.

Interestingly, from the optical linear response, shown here by the absorption coefficient (Figure 4) and the refractive index (Figure 5), it can be noticed that zinc blende BAs is isotropic from the optical point of view concerning the analysis of Γ–Γ transitions, regardless of the assumed BSE or IPA simulation schemes. Additional results similar to Figures 4 and 5 are presented in Figures S11–S16 of the Supporting Information^40^ for different applied hydrostatic pressure amplitudes, where Figures S15 and S16 show the combined version of Figures 4 and 5, collapsing the absorption coefficients and the BAs refractive indexes taking different pressures. It should be noted that the *xx*, *yy*, and *zz* absorption coefficients, due to linear light polarization along the *x*, *y*, and *z* directions, respectively, at each approach level of calculation with (BSE – solid curves) or without (IPA – dashed curves) exciton effects are rough equivalents. The isotropic nature of the optical responses lies in the fact that at the Γ–Γ energetic difference regime, the conduction and valence bands at the Γ-point present an isotropic parabolic band dispersion along the Γ-*X* and Γ-*W* paths, and the dielectric function is a macroscopic second-rank tensor; consequently, any optical response coming from the band structures would also exhibit an isotropic character. It should be noted that when considering exciton effects at 0 GPa (Figure 4a), one observes a slight deviation between *xx* results with respect to *yy* and *zz*, whose minor deviation of isotropy must have arisen due to computational errors or from the linear combination of electron–hole pairs. As expected, given Γ–Γ transitions, the absorption coefficient is null for photon energy values below the energetic difference at the Γ-point (Δ*E*_Γ_), i.e., for photon energies below 4.13 eV for 0 GPa; 3.50 eV for 44 GPa; 3.31 eV for 88 GPa; and 3.32 eV for 103 GPa. For photon energies (*ℏ*ω) higher than the energetic difference at the Γ-point (Δ*E*_Γ_), the absorption coefficients are enhanced, described in a simple picture by the following proportionality equation . Moreover, the optical band gap represented by the direct band gap (direct exciton ground state) at the IPA (BSE) reduces as the pressure increases, in agreement with the HSE06-based direct Γ–Γ band gap calculations and band structures shown in Figure 3 (red open circles) and band gap values depicted in Table 1. It should be noted in Figure 4 that the difference between the IPA and BSE optical band gaps is not clearly resolved in Figure 4 due to the small exciton binding energy. The excitonic effects in the absorption spectra can be summarized by a slight redshift for the lowest photon energies, leading to a higher absorption coefficient within the BSE scheme when compared with the IPA scheme for photon energies below 6 eV. This value corresponds to the BSE and IPA crossing point when the absorption coefficient computed within the IPA scheme becomes higher than that calculated within the BSE scheme. It is worth mentioning that if one assumes the indirect band gap transition scenario instead of the direct band gap regime displayed in Figure 4, it would expect that the absorption coefficient should scale with the square of the indirect band gap (*E*_g_^*i*^), such as^26^ α ∝ (*ℏ*ω – *E*_g_^*i*^)^2^. Furthermore, the presence of defects and impurities in the bulk BAs creates additional channels for optical transitions, leading to enhancement of the absorption coefficient, especially at photon energies smaller than the indirect band gap, i.e., it makes to appear middle gap defect states in the BAs band structure and consequently an additional α_def_ absorption defect-induced contribution.^23,26,55,56^ For the direct Γ–Γ transition regime, our results in Figure 4 show a good agreement with the experimental results by Song and co-workers in ref (26) for the direct band gap transition case. For the indirect transition regime in systems where the CBM at the *X*-point overlaps with the VBM at the Γ-point, the material would exhibit metallic behavior and, in turn, would result in metallic screening effects that suppress excitonic phenomena and introduce Drude-like free electron absorption, which is not captured by the BSE formalism. Therefore, the interpretation of excitonic features in such a regime should be treated with caution since excitonic contributions may not be valid once the system approaches a metallic or semimetallic state due to the band overlap. Here, it was explored solely the optical properties related to the direct Γ–Γ transitions.

In Figure 5, we show the refractive index as a function of photon energy, ranging from 0 to 12 eV. It indicates the relationship between the speed of light through a given material and in the vacuum medium. The calculations were evaluated for applied hydrostatic pressure magnitudes ranging from 0 to 103 GPa within the BSE (solid curves) and IPA (dashed and dotted curves) simulation schemes. As shown in Figure 5a at zero pressure, the maximum refractive index reaches the value of 4.59 in the visible region within the BSE simulation level for the photon energy around 4.40 eV. For non-null applied hydrostatic pressure, Figure 5b–d shows that the peak of the refractive index presents a slight redshift. Interestingly, owing to the application of hydrostatic pressure, the maximum magnitude of the refractive index peak decreases, whereas for near-zero photon energy, the refractive index increases from 2.61 for 0 GPa to 2.67 for 44 GPa, to 2.72 for 88 GPa, and to 2.75 for 103 GPa. By comparing the IPA and BSE results in Figure 5, one notices a weak contribution of excitonic effects on the magnitude of the refractive index, as also reported in ref (26).

## 4 Conclusions

In summary, by employing first-principles calculations combining different schemes, such as the MLWF-TB model and BSE formalism, we have presented a systematic and carefully taken study on the effects of hydrostatic pressure on the physical properties of bulk zinc-blended BAs, such as band gap engineering and their consequences on the excitonic and optical properties. Concerning the BAs’s electronic properties, we have shown that (i) the hydrostatic pressure reduces the band gap in a quasi-linear-like form when the pressure increases due to the contraction of the crystalline lattice; (ii) a three-fold degeneracy breaking occurs at the VBM when the applied hydrostatic pressure increases; (iii) the semiconductor-to-metal transition happens without any crystallographic phase transition for the pressure range examined, in accordance with the results in the literature; and (iv) the HSE06 band gap (1.73 eV) is consistent with the experimental band gap of 1.45–2.07 eV.^12,17,25−27^ Based on our analyses of the excitonic effects on the optical properties of BAs, we have found that the absorption coefficients and refractive indexes of the bulk BAs (i) remain isotropic under a non-null applied hydrostatic pressure; (ii) exhibit a pressure-induced redshift in their spectra; and (iii) demonstrate small excitonic contributions when one compares the BSE and IPA results. Our results presented here exhibited compatibility in the electronic and optical studies, including that BAs operates within the wavelength range of the visible spectrum under pressure effects.

Due to the fact that semiconductor-based systems are the main building blocks of nanoelectronics devices and many technological applications demand band gap controlling, we believe that the theoretical results presented in our investigation can be useful for a better understanding of the relevant physics of the cubic BAs subjected to hydrostatic pressure as a way to achieve band gap control and the tuning of their optoelectronic and excitonic properties.

## Supporting Information Available

Description of atomic structures, on-site energy contributions in our tight-binding model, electronic properties, optical properties; crystallographic structure information; pressure dependence of bulk volume; site energies for different pressures; band structures; and projected density of states (PDF)
