# Supplementary material for: Electronic, Excitonic, and Optical Properties of Zinc Blende Boron Arsenide Tuned by Hydrostatic Pressure
Source: ACS Omega. 2024 Nov 16;9(48):47710–8. doi: 10.1021/acsomega.4c07598 (PMC11618452; doi:10.1021/acsomega.4c07598)
Supplement: Supplementary file 1 — ao4c07598_si_001.pdf [file ao4c07598_si_001.pdf]

Supporting Information: Electronic, excitonic and optical properties of zinc blende boron arsenide tuned by hydrostatic pressure

Elisangela da Silva Barboza,[†] Alexandre C. Dias,[‡] Luis Craco,[†] Sabrina S. Carara,[†] Diego R. da Costa,[*,¶,§] and Teldo A. S. Pereira[*,†,‖]

†*Instituto de Física, Universidade Federal de Mato Grosso, 78060-900, Cuiabá, MT, Brazil*

‡*Institute of Physics and International Center of Physics, University of Brasília, Brasília 70919-970, DF, Brazil*

¶*Departamento de Física, Universidade Federal do Ceará, 60455-900, Fortaleza, CE, Brazil*

§*Department of Physics, University of Antwerp, Groenenborgerlaan 171, B-2020 Antwerp, Belgium*

‖*National Institute of Science and Technology on Materials Informatics, Campinas, Brazil*

E-mail: diego_rabelo@fisica.ufc.br; teldo@fisica.ufmt.br

Contents

1 **Atomic Structures** **S-2**

2 **On-site energy contributions in our tight-binding model** **S-4**

3 **Electronic Properties** **S-6**

4 **Optical Properties** **S-13**

References **S-19**

1 Atomic Structures

According to Table S1 and Fig. S1, one can confirm that under hydrostatic pressure, the volume, atomic positions, and lattice parameters decrease as pressure increases, while the angles between the lattice vectors remain constant, maintaining the cubic symmetry of the material. This is consistent with the behavior expected under hydrostatic pressure, where the material contracts isotropically. Furthermore, to provide additional verification, we depict in Fig. S2 images of the crystal structure obtained via XCrySDen after relaxation under pressure. It allows us to visually confirm that the symmetry of the crystal structure is maintained throughout the relaxation process. As shown in Fig. 1(d) of the manuscript, similarly in Fig. S1, and also supported by literature References[S1,S2] (Refs. [12] and [33] of the manuscript), our results for the ratio (V/V_0) under pressure show a good agreement with experimental data.

For Fig. 1(d) (see Fig. S1), we used the Birch-Murnaghan equation for a better fit, while for Fig. 3(e) (see Fig. S6), we opted a linear adjustment. The best fit for the volume versus pressure data, in comparison with experimental results, is the fit using the Birch-Murnaghan equation. Therefore, the equation used for the fit was:

$$\frac{V(P)}{V_0} = \left(1 + \frac{3}{2}\left(\mathrm{B}_0^{'} - 1\right)\left(\frac{P}{B_0}\right)^{2/3}\right). \quad (1)$$

The data related to the Bulk Modulus values B_0 and the derivative of the Bulk Modulus B_0'

are determined by fitting the curve-fit function from the SciPy package in Python. In this way, the curve-fit-function uses numerical minimization methods to determine these values. This code reads the experimental data, applies the Birch-Murnaghan equation fit, and provides appropriate curves with our data and experimental results from References[S1,S2]. The fitted equation for each dataset is as follows:

- This Work fit:

$$\frac{V(P)}{V_0} = \left(1 + \frac{3}{2}(0.99 - 1)\left(\frac{P}{0.85}\right)^{2/3}\right). \tag{2}$$

- Fit of Reference[S1] (similar as Ref. [12] in the main text):

$$\frac{V(P)}{V_0} = \left(1 + \frac{3}{2}(0.99 - 1)\left(\frac{P}{0.84}\right)^{2/3}\right). \tag{3}$$

- Fit of Reference[S2] (similar as Ref. [33] in the main text):

$$\frac{V(P)}{V_0} = \left(1 + \frac{3}{2}(0.99 - 1)\left(\frac{P}{1.12}\right)^{2/3}\right). \tag{4}$$

Table S1: Crystallographic structure information of boron arsenide (BAs) under different hydrostatic pressures applied to the material, such as: the B-As bond distances (second column), the angles between atomic sites (third column), and the lattice parameters of the crystal (fourth column).

Pressure (GPa)	Distance (B-As) (Å)	Angle (θ)(B-As-B)	Parameter (Å)
0	2.065	109.471	4.77
14	2.023	109.471	4.67
29	1.977	109.471	4.56
44	1.941	109.471	4.48
59	1.911	109.471	4.41
74	1.885	109.471	4.35
88	1.863	109.471	4.30
103	1.843	109.471	4.26
118	1.825	109.471	4.21

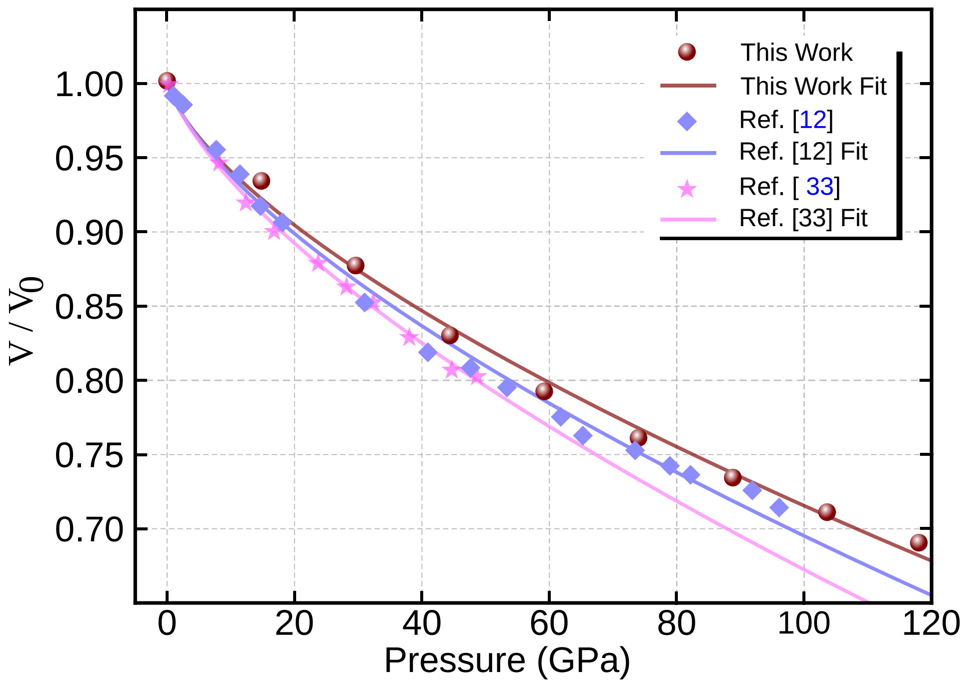

Figure S1: Pressure dependence of bulk volume (V) for the BAs crystal normalized to the value obtained under ambient pressure conditions (V_0). Our results (brown spheres) are compared with experimental values taken from Refs.[S1,S2] (similar to Refs. [12] and [33] in the main text), showing very good agreement between theory and experiment. The data was obtained using the Birch-Murnaghan search to make this fit.

2 On-site energy contributions in our tight-binding model

In our TB model, derived from maximally localized Wannier functions (MLWFs) using the Wannier90 package, we used 8 orbitals to compose the basis set: s, p_x, p_y, and p_z orbitals from both B and As atoms. The TB Hamiltonian matrix contains the on-site energies and hopping terms, with 4497 hopping vectors representing the interaction between the central unit cell and neighboring cells. These hopping terms account for the orbital overlap between B and As atoms, capturing the reduced ionicity and the shared character of the valence bands.

Regarding the on-site energies of the orbitals, they reflect the proximity of the B and As p levels and the small ionicity. For example, the on-site energies for the B p orbitals are very close to those of the As p orbitals (see Table S2). This indicates that neither atom dominates the valence band contribution, supporting the notion that the roles of the group III and group V elements are almost reversed. These on-site energy elements and their implications on the electronic structure of the material reflect on the mixed character of B p-like and As p-like states

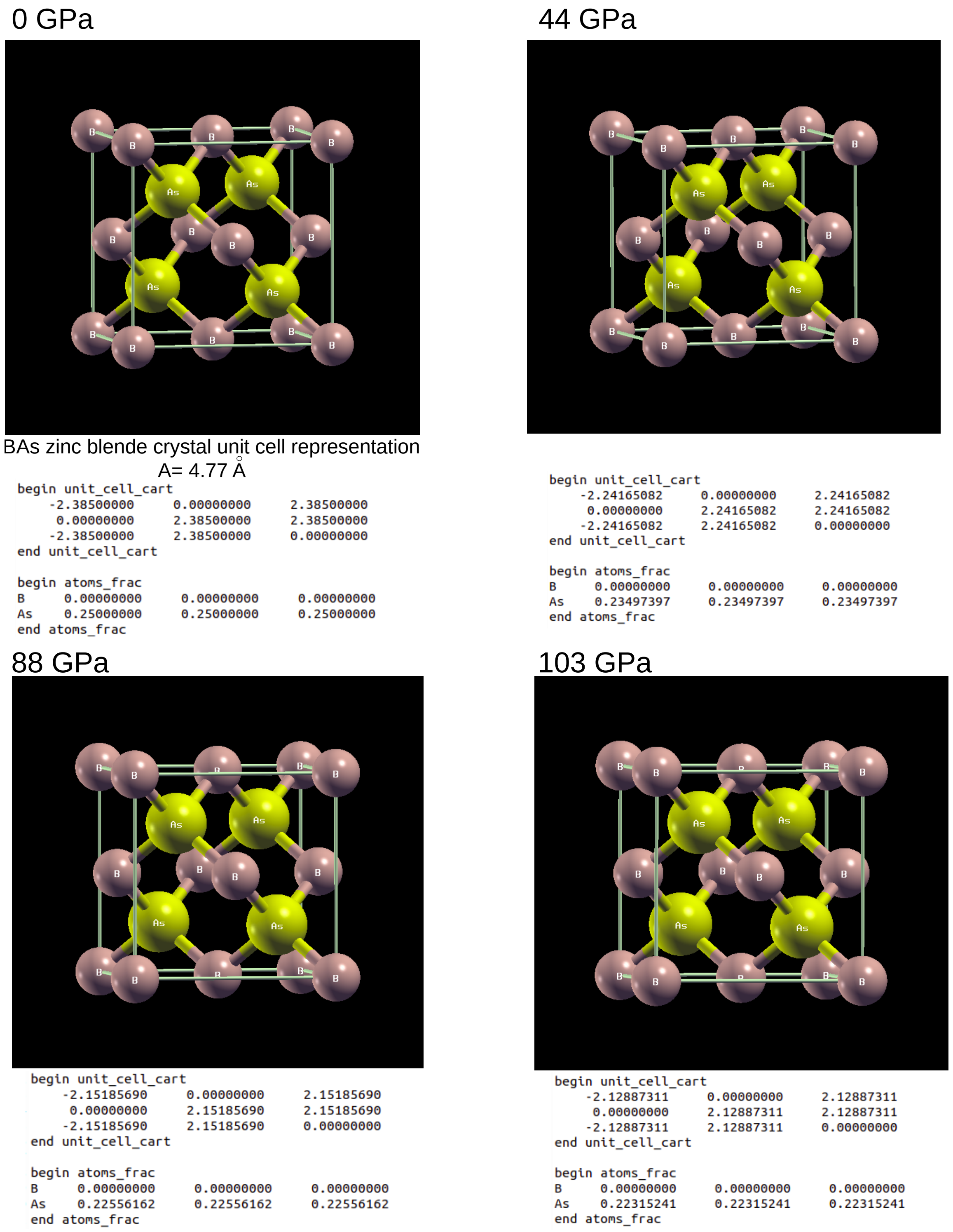

Figure S2: Representation of the optimized unit cell of the BAs crystal in the zinc-blende structure under various pressures, using the XCrySDen application. The caption below each figure shows the lattice vectors provided in angstroms (Å), as well as the corresponding atomic positions of each atom in the BAs crystal in fractional units.

of the valence bands, as can be verified in Fig. 2 of the main manuscript and also in Figs. S8 and S10 of the present Supplementary Material.

Table S2: Site energies for different pressures (0 GPa, 44 GPa, 88 GPa, and 103 GPa) with Fermi level values. The first column corresponds to the site energy associated with the *s* orbital, while the other columns correspond to the *pz*, *px*, and *py* orbitals, respectively.

Pressure	eFermi (eV)		Site energy(*s*)	Site energy(*pz*)	Site energy(*px*)	Site energy(*pz*)
**0 GPa**	**8.7672**	B	2.526838	10.0793	10.0793	10.0793
		As	0.963346	11.509957	11.509957	11.509957
**44 GPa**	**11.9894**	B	5.7831	12.5376	12.5377	12.5376
		As	4.0035	14.085	14.085	14.085
**88 GPa**	**14.3357**	B	8.0857	14.2210	14.2210	14.2210
		As	6.7126	15.9945	15.9945	15.9945
**103 GPa**	**14.9610**	B	8.7398	14.6895	14.6895	14.6895
		As	7.4860	16.5175	16.5175	16.5175

3 Electronic Properties

As discussed in the main text, the symmetry of the BAs crystal structure is preserved under purely hydrostatic pressure, such that this homogeneously applied pressure reduces the volume but keeps the cubic symmetry of the system. This means that no symmetry has been broken, as can be seen from the results of Sec. S1. Note that (i) the angles in Table S1 are kept the same; (ii) the crystal structure obtained via XCrySDen images in Fig. S2 after relaxation under pressure is preserved cubic; (iii) the volume decreases as the pressure is applied as shown in Fig. S1.

After a detailed analysis of the BAs band structures, making an enlargement of the energy bands close to the valence band in the vicinity of the Γ-point, it can be noticed that the energies are not degenerate. Instead, the "three-fold degeneracy" in the band structure of Fig. 2 of the main text at $P = 0$ GPa was only apparent and not real degenerate. See Fig. S3 that shows a zoom of the valence bands for four applied hydrostatic pressure amplitudes: (a) 0, (b) 44 GPa, (c) 88 GPa, and (d) 103 GPa. See Sec. IIIA for more discussions.

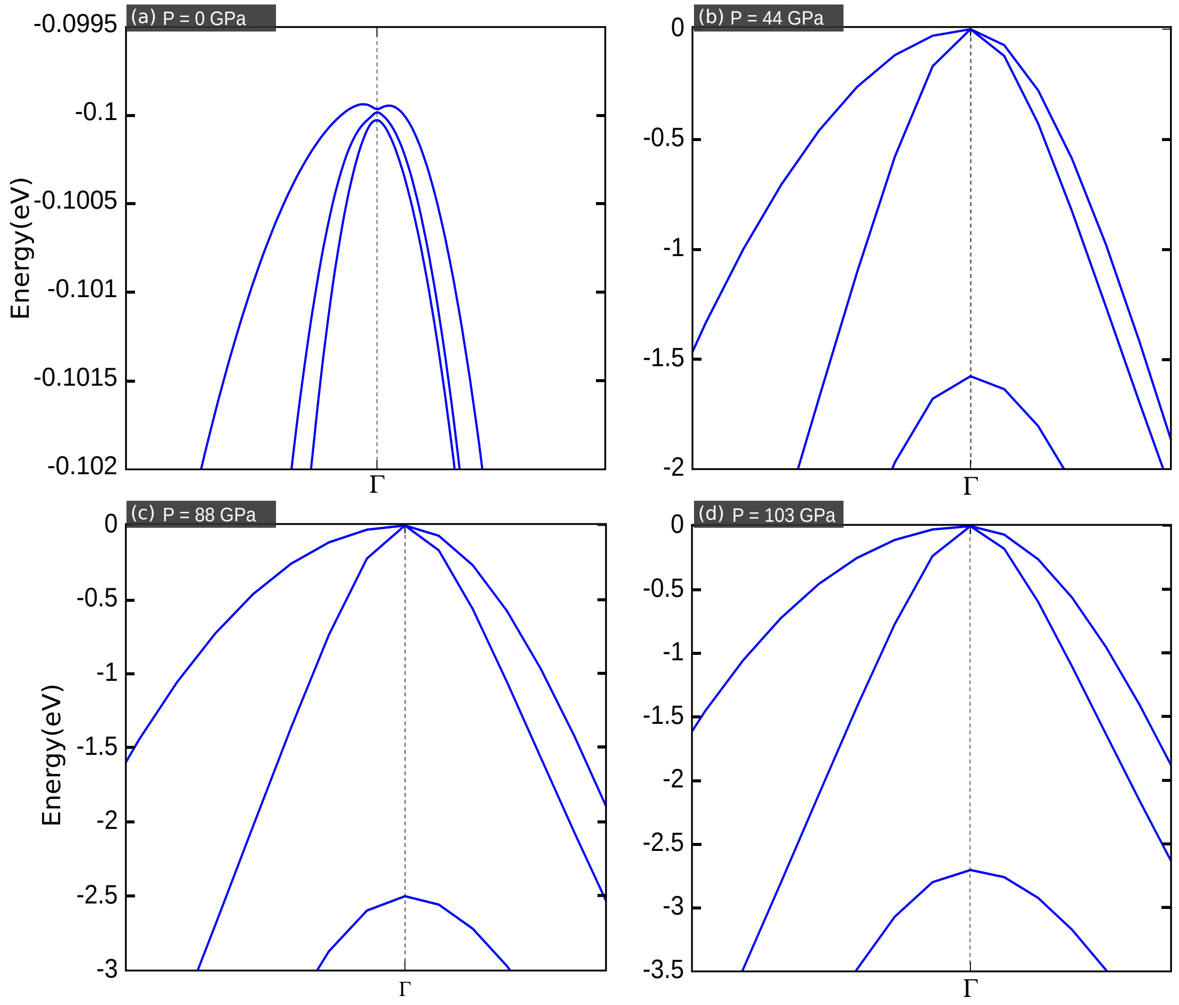

Figure S3: An enlargement of the BAs band structures around the three valence bands in the vicinity of the Γ-point for four applied hydrostatic pressure amplitudes: (a) 0, (b) 44 GPa, (c) 88 GPa, and (d) 103 GPa.

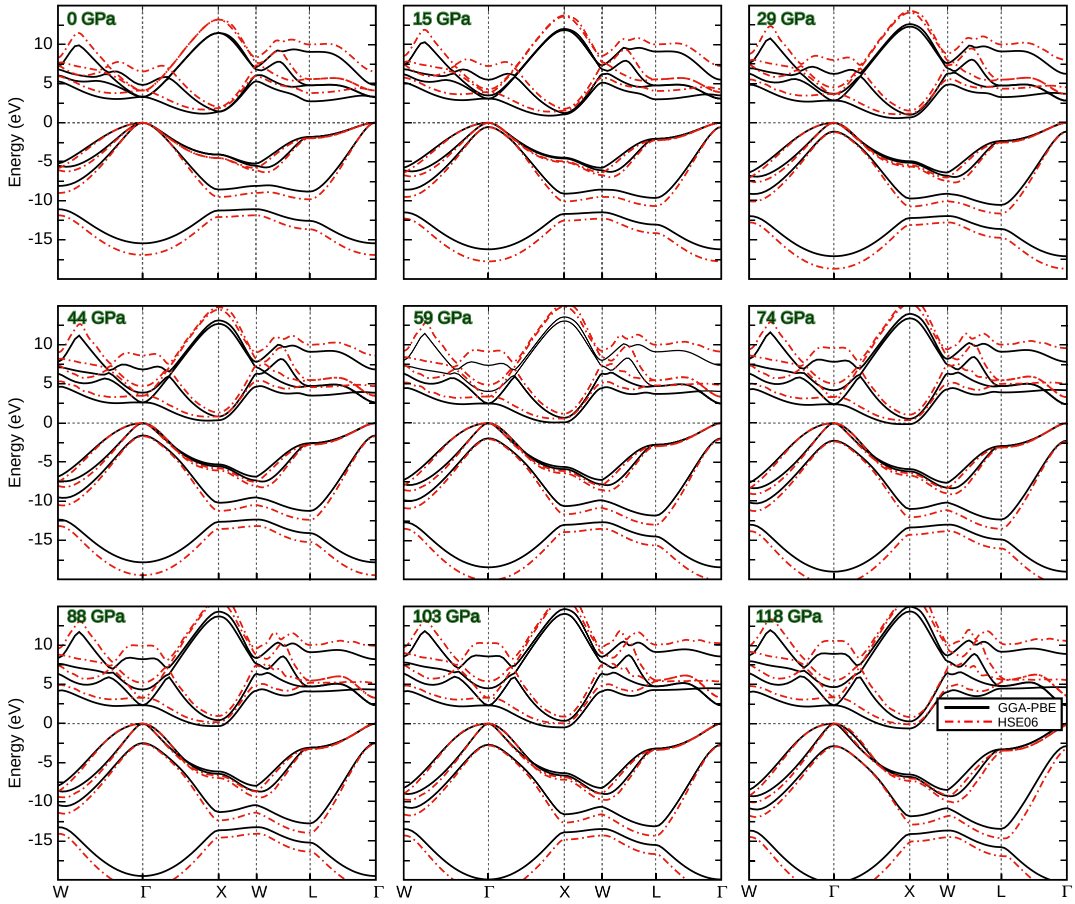

Figure S4: Band structure for various amplitudes of applied hydrostatic pressure, ranging from 0 GPa to 118 GPa. Additionally, two functionals, GGA-PBE and HSE06, were used. The band structures corresponding to the PBE functional are represented by solid black curves, while those of the hybrid HSE06 functional are denoted by dashed red curves.

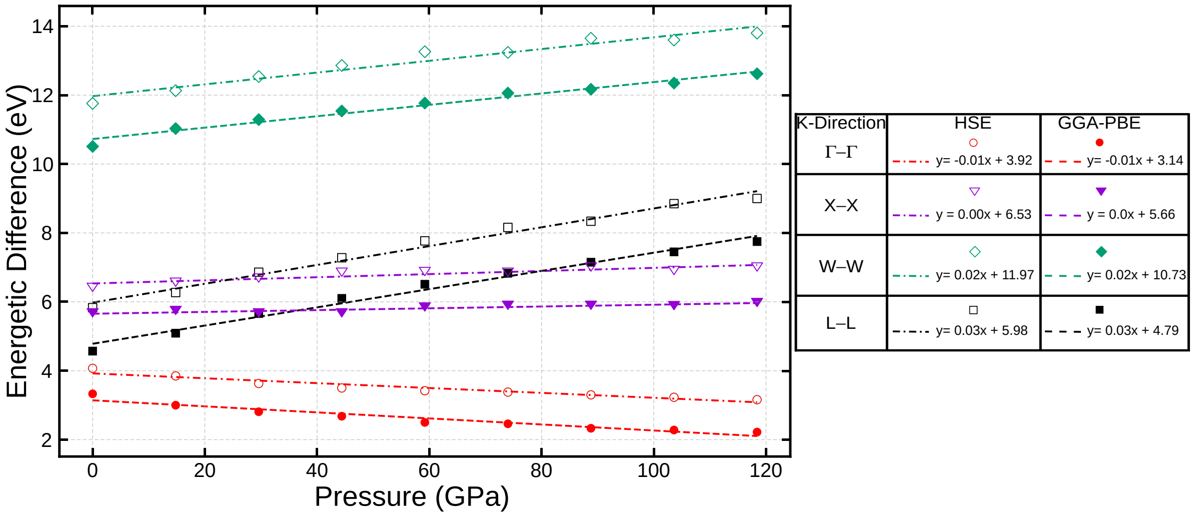

Figure S5: Energetic difference as a function of the applied hydrostatic pressure by an energetic difference from Γ to Γ, from X to X, and from W to W high symmetry points with the linear fitting of the curves over the pressure range.

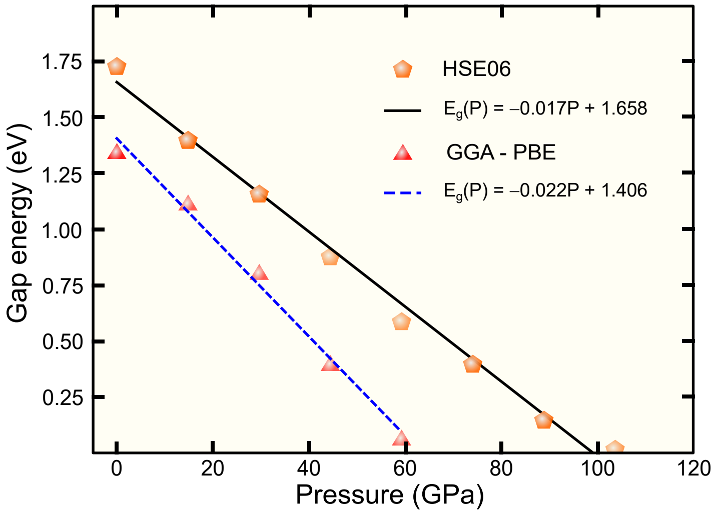

Figure S6: Band gap energy as a function of applied hydrostatic pressure, taking the real indirect band gap energy of the system, *i.e.*, for an energy difference between the VBM at point Γ and the CBM at point X, with the linear fitting of the curves over the pressure range.

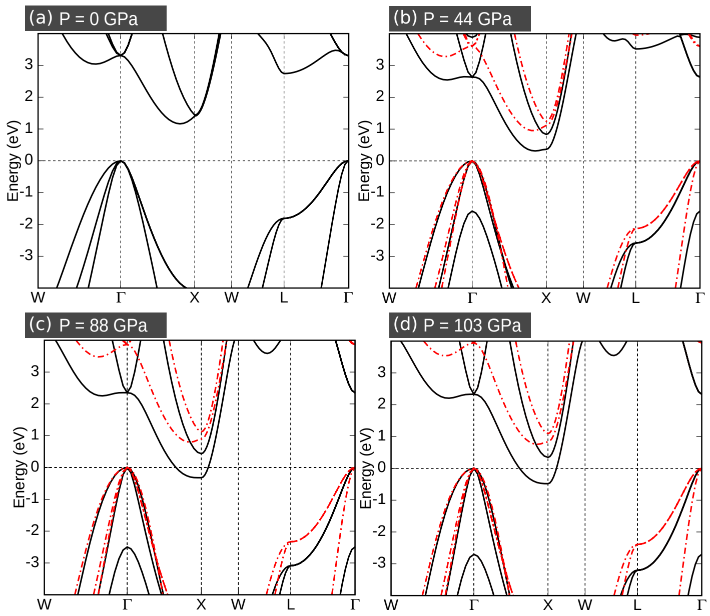

Figure S7: Band structures of BAs, computed using the PBE approximation, under different hydrostatic pressures: (a) 0 GPa, (b) 44 GPa, (c) 88 GPa, and (d) 103 GPa. The solid black curves correspond to the results varying As positions, while the dashed red curves correspond to the configuration assumed throughout the paper by fixing the relative atomic positions.

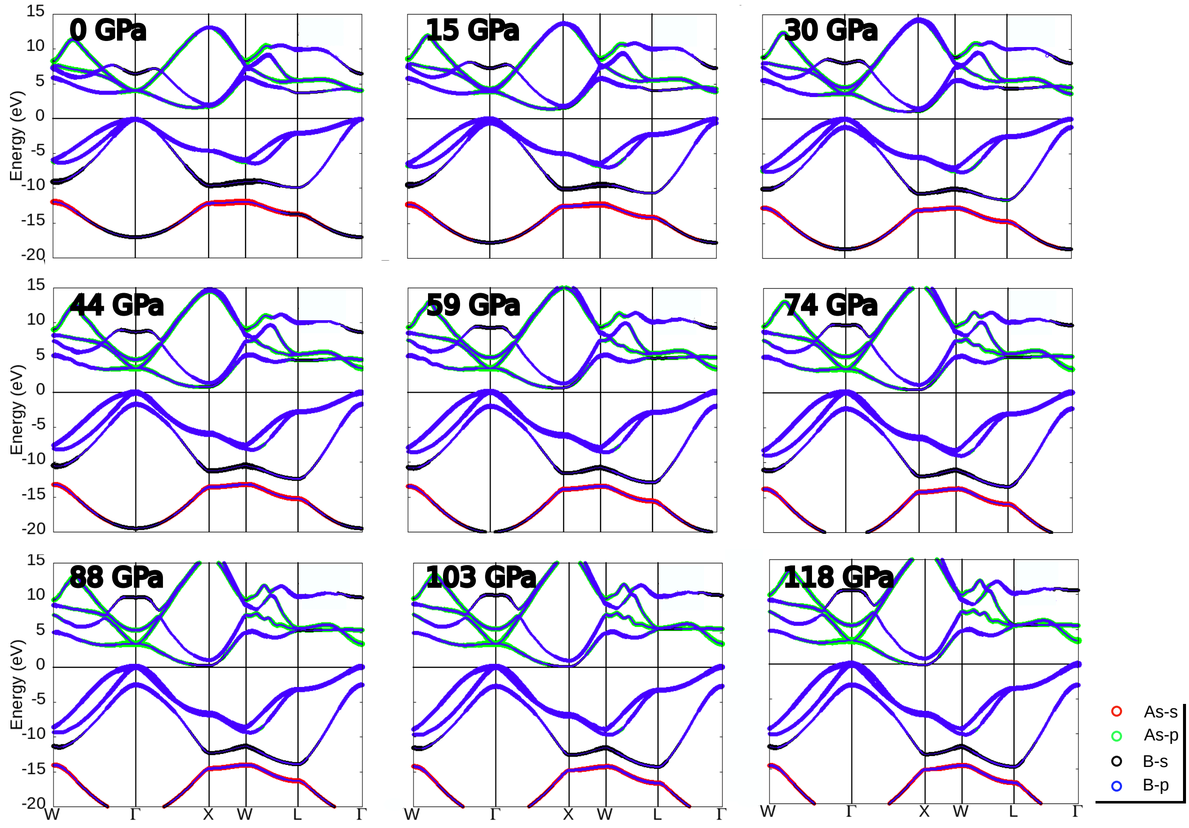

Figure S8: Band structures with projected orbitals using the HSE06 functional for various amplitudes of applied hydrostatic pressure. For arsenic (As), the red circle represents the s orbital, and the green circle denotes the p orbital. For the boron (B) atom, the s orbital is indicated by the black circle, and the p orbital by the blue circle.

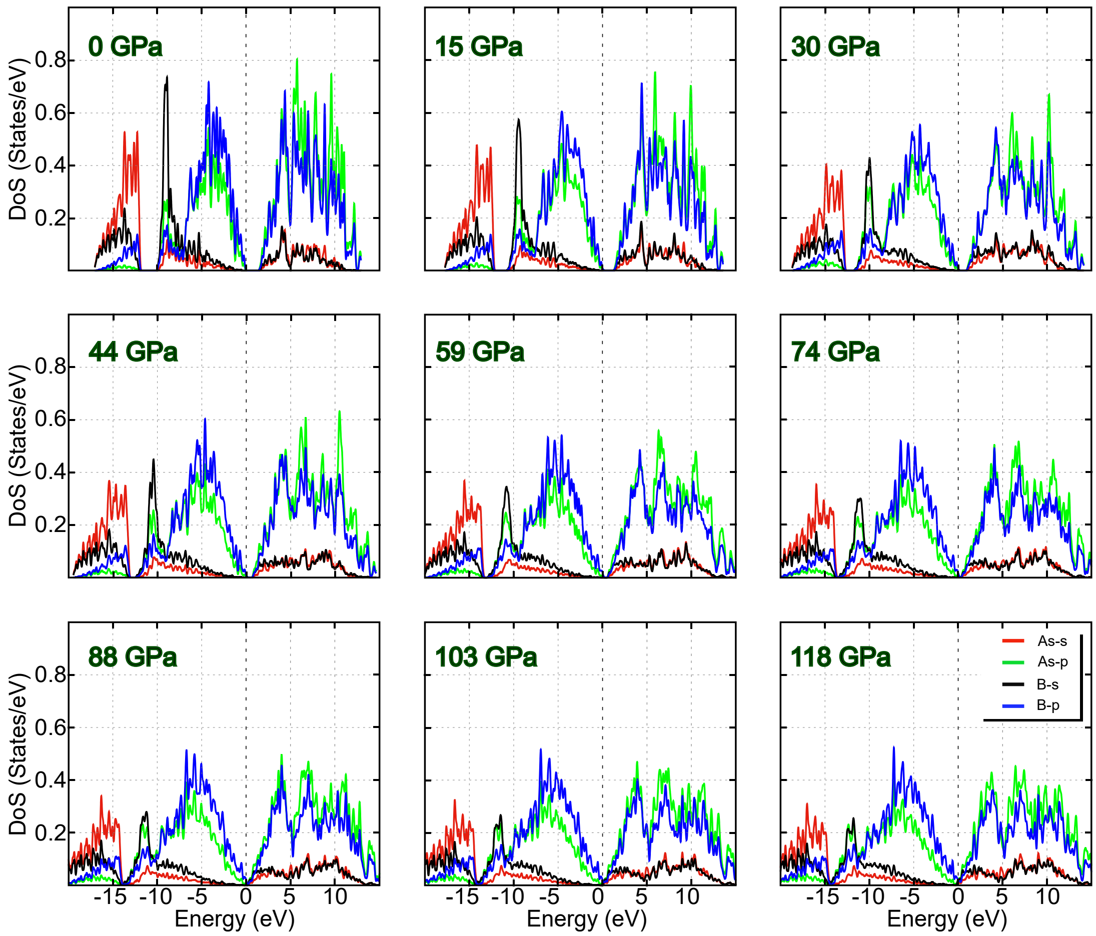

Figure S9: Projected density of states for various amplitudes of applied hydrostatic pressure. The red line represents the s orbital of the arsenic (As) atom, and the green line corresponds to the p orbital of As. For boron (B), the black line corresponds to the s orbital, and the blue line refers to the p orbital.

Table S3: 3D effective mass tensor of boron arsenide (BAs) for electrons, denoted as m_{ijk} with **i, j, k** $\equiv x, y, z$ in units of the electron rest mass m_0.

Press (GPa)	m_{xx}	m_{yy}	m_{zz}	m_{xy}	m_{xz}	m_{yz}
0 GPa	1.6481	0.2207	0.2207	168.158	165.157	30.2013
14 GPa	2.1140	0.2336	0.2314	28.0612	41.3358	1.8162
29 GPa	1.7258	0.2201	0.2171	3.9247	4.2620	0.7729
44 GPa	1.1362	0.2101	0.2120	5.8580	5.2426	0.4946
59 GPa	1.9957	0.2008	0.2003	17.7777	13.5930	0.3805
74 GPa	1.8782	0.1859	0.1872	6.6070	8.7324	0.3196
88 GPa	8.1064	0.1945	0.1949	19.5067	16.0172	0.3000
103 GPa	2.5294	0.1949	0.1958	5.9163	5.3303	0.2681
118 GPa	17.5827	0.1971	0.1941	9.5699	18.6365	0.2937

Table S4: 3D effective mass tensor of boron arsenide (BAs) for holes, denoted as m_{ijk} with **i, j, k** $\equiv x, y, z$ in units of the electron rest mass m_0.

Press (GPa)	m_{xx}	m_{yy}	m_{zz}	m_{xy}	m_{xz}	m_{yz}
0 GPa	0.3212	0.5668	0.3408	6.2913	0.6485	0.9028
14 GPa	0.1816	0.2336	0.2506	2.1858	1.9613	0.2298
29 GPa	0.1614	0.2435	0.2138	0.9858	0.6281	0.2432
44 GPa	0.1860	0.2242	0.1749	0.2786	1.0023	0.5061
59 GPa	0.1797	0.2602	0.2859	0.7805	0.5926	0.3281
74 GPa	0.1637	0.2637	0.1582	0.3010	1.4072	0.3486
88 GPa	0.2364	0.2271	0.2004	0.2535	1.3096	0.3116
103 GPa	0.2376	0.2072	0.1533	0.2506	1.4173	0.3040
118 GPa	0.2965	0.2832	0.1466	0.3163	0.8173	0.3204

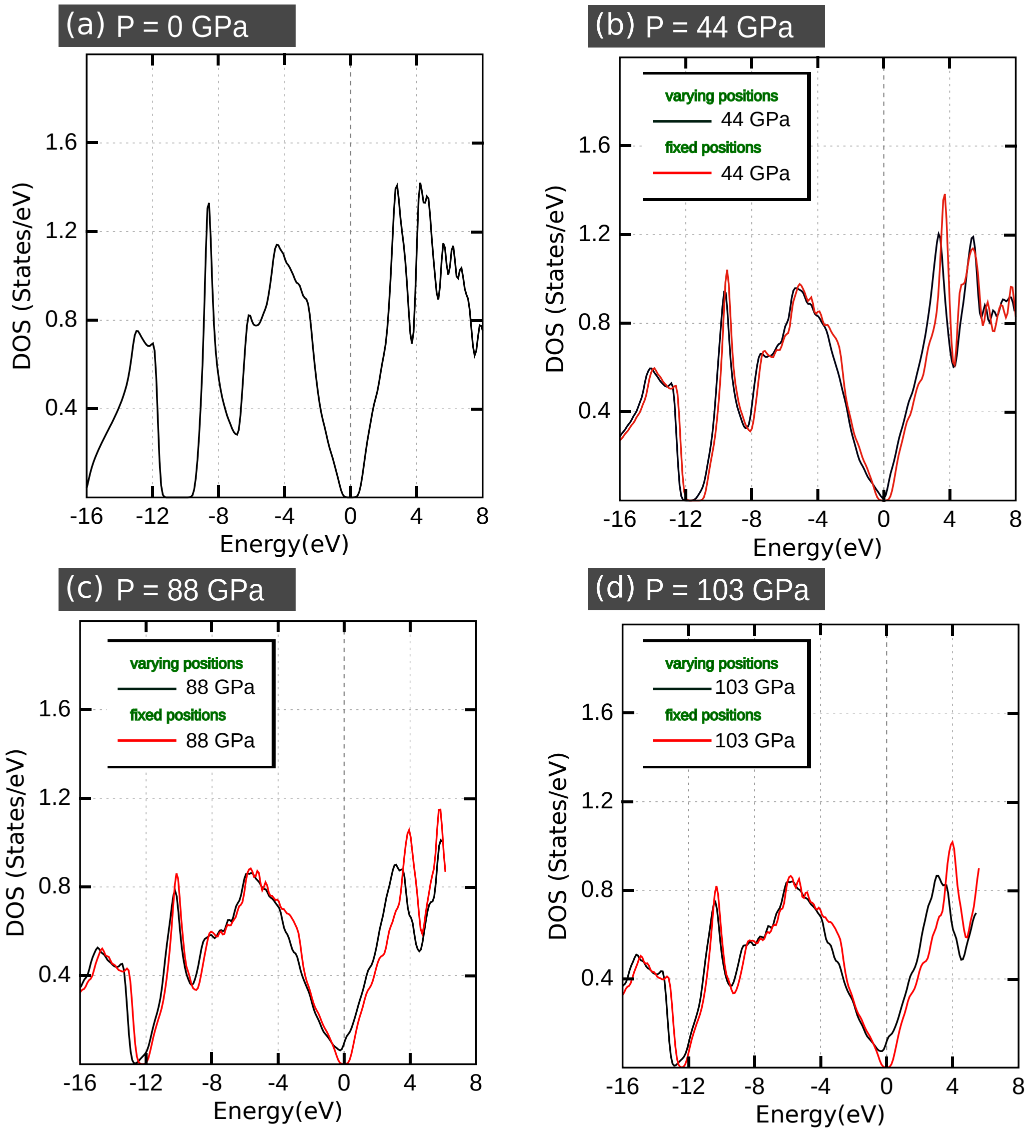

Figure S10: Total density of states for various amplitudes of applied hydrostatic pressure. The black curve corresponds to the results in which one assumes that the positions of the arsenic atoms (As) vary with pressure, whereas the red curve corresponds to the fixed As atom $(1/4, 1/4, 1/4)$ case.

4 Optical Properties

The refractive index was computed using the WanTiBEXOS computational package[S3] obtained from the real ($\varepsilon_{1,\alpha,\beta}$) and imaginary ($\varepsilon_{2,\alpha,\beta}$) parts of the dielectric function by the following expression:

$$n_{\alpha,\beta}(\omega) = \left[\frac{\sqrt{\varepsilon^2_{1,\alpha,\beta}(\omega) + \varepsilon^2_{2,\alpha,\beta}(\omega)} + \varepsilon_{1,\alpha,\beta}(\omega)}{2} \right]^{\frac{1}{2}}, \tag{5}$$

where α and β correspondent of the dielectric tensor components. The smearing used was intended to improve the plotted results to better match the experimental data. Additionally, to further improve the results, it would be necessary to use a denser k-mesh, making the calculation computationally expensive.

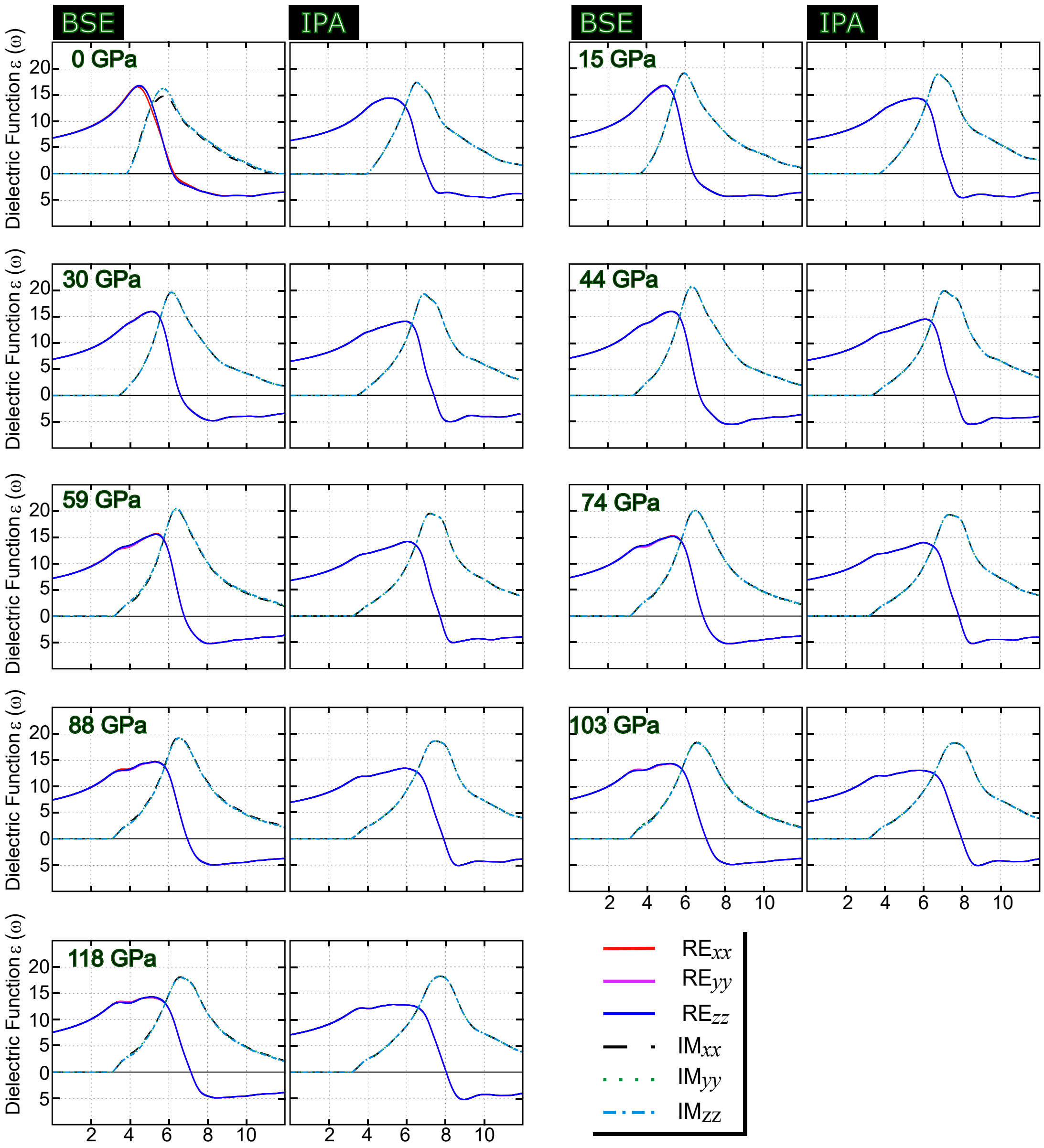

Figure S11: Optical property related to the real and imaginary dielectric function obtained through the MLWF-TB approach at both the BSE level (solid curves) and the IPA level (dashed curves) for various applied hydrostatic pressure amplitudes and different light polarizations.

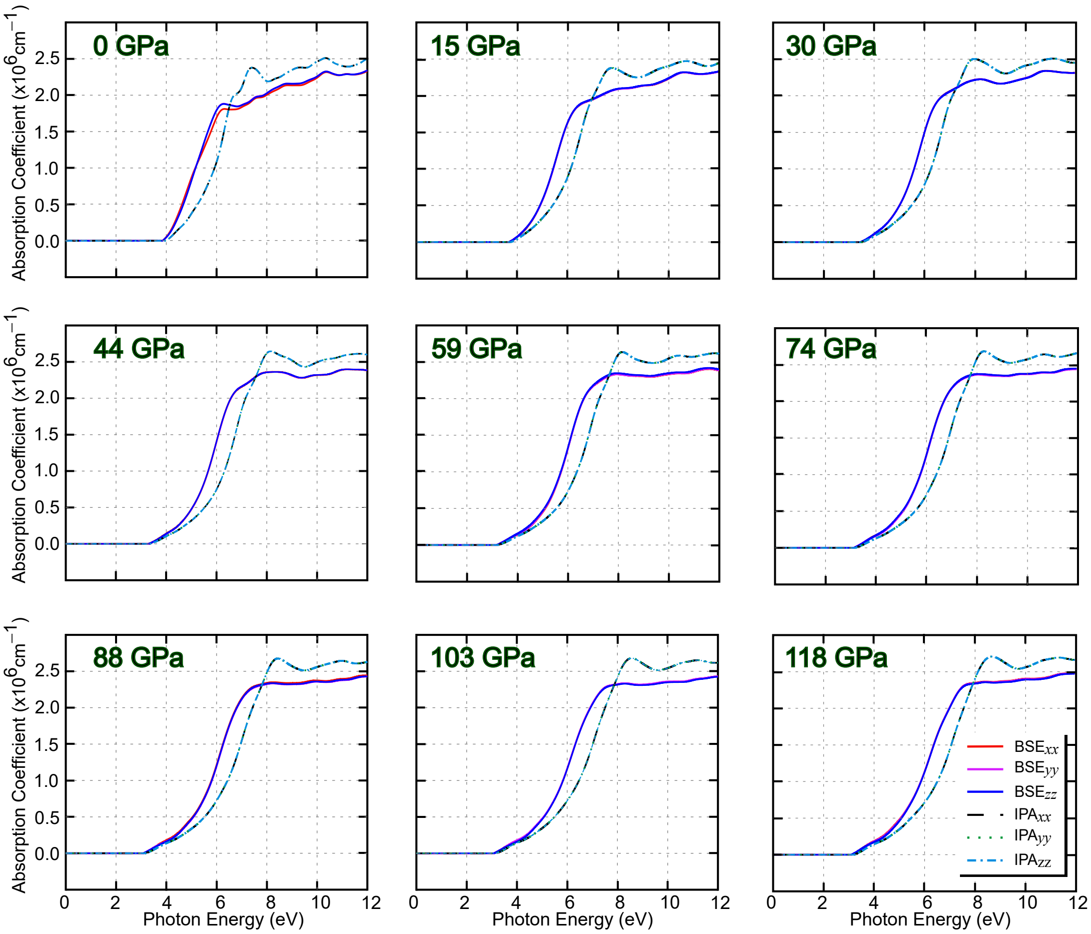

Figure S12: Optical property related to the absorption coefficients obtained through the MLWF-TB approach at both the BSE level (solid curves) and the IPA level (dashed curves) for various applied hydrostatic pressure amplitudes and different light polarizations.

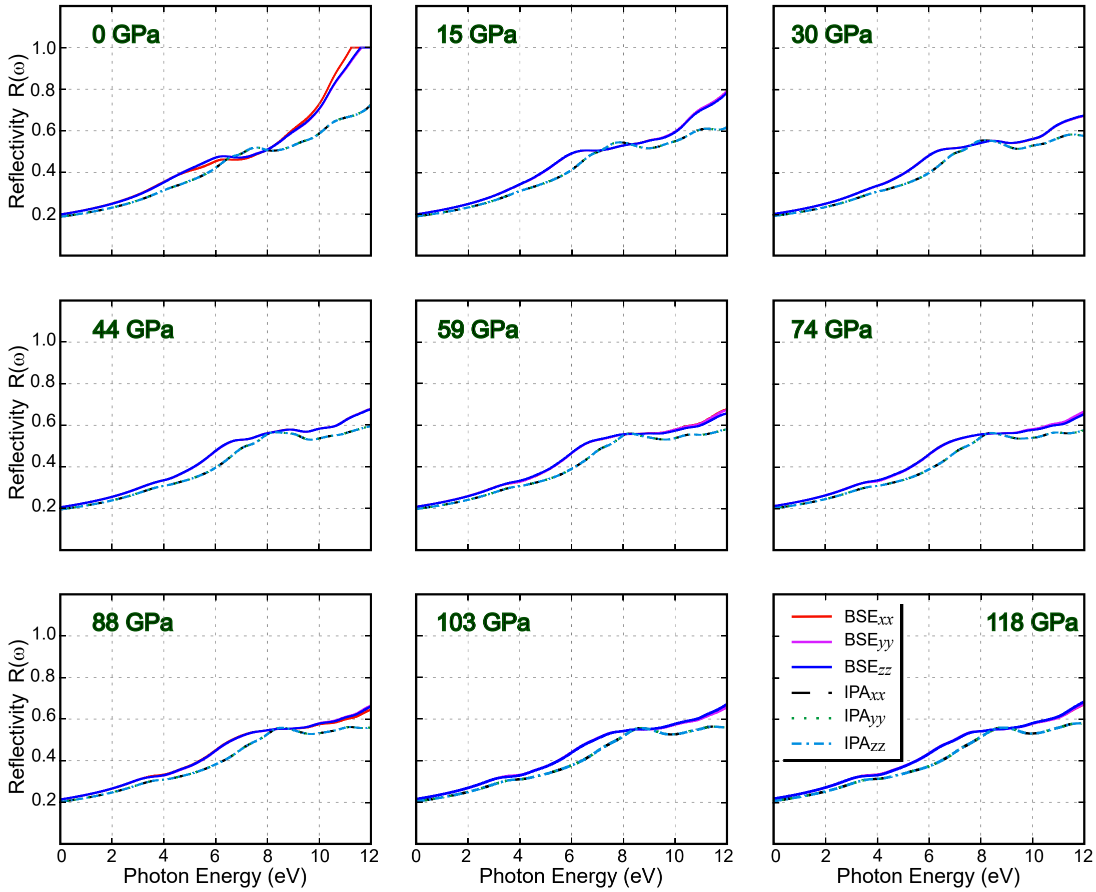

Figure S13: Optical property related to the total reflectivity obtained through the MLWF-TB approach at both the BSE level (solid curves) and the IPA level (dashed curves) for various applied hydrostatic pressure amplitudes and different light polarizations.

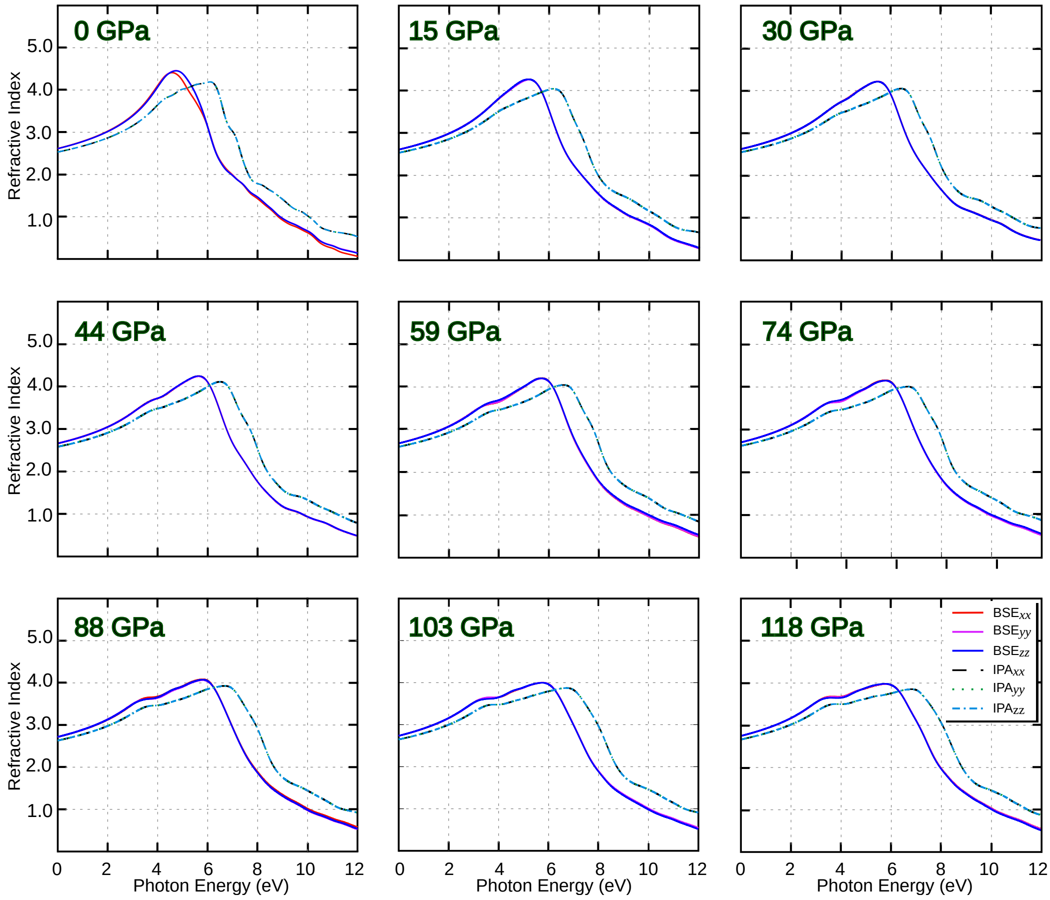

Figure S14: Optical property related to the total refractive indices obtained through the MLWF-TB approach at both the BSE level (solid curves) and the IPA level (dashed curves) for various applied hydrostatic pressure amplitudes and different light polarizations.

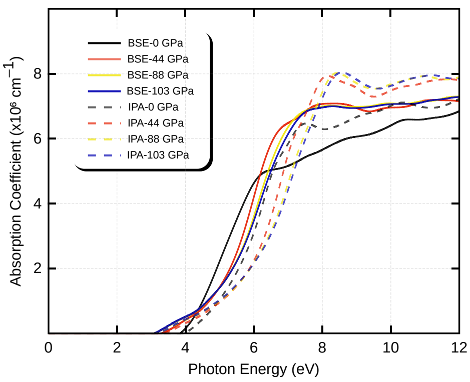

Figure S15: BAs absorption coefficients computed within the BSE (solid curves) and IPA (dashed curves) simulation schemes, considering the light polarization at x, y and z directions for different hydrostatic pressures: (black curves) 0 GPa, (red curves) 44 GPa, (yellow curves) 88 GPa, and (blue curves) 103 GPa.

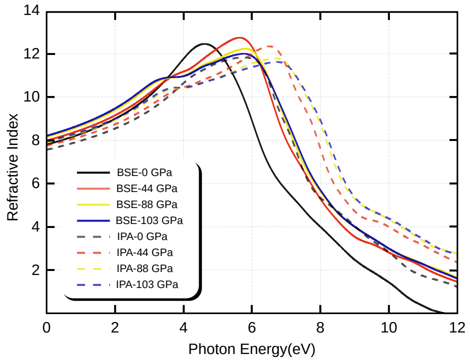

Figure S16: BAs refractive indexes obtained using BSE (solid curves) and IPA (dashed curves) simulation schemes, considering the light polarization at x, y, and z directions for different hydrostatic pressures: (black curves) 0 GPa, (red curves) 44 GPa, (yellow curves) 88 GPa, and (blue curves) 103 GPa.

References

(S1) Tian, F.; Luo, K.; Xie, C.; Liu, B.; Liang, X.; Wang, L.; Gamage, G. A.; Sun, H.; Ziyaee, H.; Sun, J.; Zhao, Z.; Xu, B.; Gao, G.; Zhou, X.-F.; Ren, Z. Mechanical properties of boron arsenide single crystal. *Applied Physics Letters* **2019**, *114*, 131903.

(S2) Greene, R. G.; Luo, H.; Ruoff, A. L.; Trail, S. S.; DiSalvo, F. J. Pressure Induced Metastable Amorphization of BAs: Evidence for a Kinetically Frustrated Phase Transformation. *Physical Review Letters* **1994**, *73*, 2476–2479.

(S3) Dias, A. C.; Silveira, J. F.; Qu, F. WanTiBEXOS: A Wannier based Tight Binding code for electronic band structure, excitonic and optoelectronic properties of solids. *Computer Physics Communications* **2023**, *285*, 108636.
